# Myoinositol Versus Metformin in the Treatment of Polycystic Ovarian Syndrome: A Systematic Review

**DOI:** 10.7759/cureus.41748

**Published:** 2023-07-11

**Authors:** Ranita Bodepudi, Saniya Seher, Shenel A Khan, Sonya Emmanuel, Vivig Shantha Kumar, Resheek Nerella, Basim Shaman Ameen, Dev Patel, Jabez David John, Safeera Khan

**Affiliations:** 1 Medicine, California Institute of Behavioral Neurosciences & Psychology, Fairfield, USA; 2 Internal Medicine, Lokmanya Tilak Municipal Medical College and General Hospital, Mumbai, IND

**Keywords:** inositol, metformin, myoinositol, pcos, polycystic ovary syndrome, stein-leventhal syndrome

## Abstract

Polycystic ovarian syndrome (PCOS) is a widespread, complex, and multi-system hormonal disorder that occurs in women of reproductive age. The wide variation in practice in the treatment of PCOS is a direct consequence of the lack of sufficient evidence on alternative treatment strategies, as well as a poor understanding of the disorder itself. The aim of our systematic review was to assess the therapeutic advantages and adverse effects of metformin (MET), a standard treatment modality, with myoinositol (MI), a recent substitute that may be used alone or in combination with other remedies to treat PCOS. A literature search was done using PubMed Central, PubMed, Medline, Cochrane, Science Direct, and Google Scholar. Studies were limited to those published in English between 2012 and 2022 that focused on the management of PCOS with both MET and MI. The systematic review complied with the Preferred Reporting Items for Systematic Reviews and Meta-Analyses (PRISMA) 2020 guidelines. Using standard quality assessment tools, two reviewers independently assessed the content of the incorporated studies. Three meta-analyses, eight randomized controlled trials (RCTs), and one non-randomized non-controlled trial (NN-RCT) were deemed eligible. Following extensive analysis, we found that MET and MI are comparable in their effects on clinical, hormonal, and biochemical profiles. MI, however, had a better safety profile and tolerance due to minimal side effects compared to MET. These results demonstrate the potential role of MI as a novel asset in the armamentarium in the management of PCOS.

## Introduction and background

Polycystic ovarian syndrome (PCOS), also known as Stein-Leventhal syndrome, is a multifaceted endocrinopathy affecting women distinguished by an array of signs and symptoms of ovarian dysfunction and androgen excess [1,2]. There are four commonly recognized salient features of PCOS: i) hyperandrogenemia, ii) ovulatory and menstrual dysfunction, iii) clinical features of hyperandrogenism, and iv) polycystic ovaries [3].

PCOS affects women of all races and ethnicities of reproductive age and is among the most common endocrinopathies, with an estimated global prevalence of 5%-15% [4,5]. World Health Organization estimates that it impacted 116 million women worldwide (3.4% of the population) as of 2010 [6].

Historically, the diagnostic criteria of PCOS have been rooted in the National Institutes of Health (NIH) consensus and have evolved over time to be now superseded by the Rotterdam criteria [7]. According to the 2003 revised Rotterdam criteria, a diagnosis of PCOS is established when two out of the three following criteria are met after ruling out all other potential conditions that mirror the PCOS phenotype: (i) clinical and/or biochemical signs of hyperandrogenism, (ii) oligo‐ and/or anovulation, and (iii) polycystic ovaries on ultrasound examination [8].

Although the precise etiology of this syndrome remains predominantly uncertain, emerging evidence indicates that PCOS might have powerful epigenetic and environmental influences, such as diet and lifestyle factors, that lend to its complex and multigenic nature [2]. Complex positive feedback of insulin resistance and hyperandrogenism characterizes the pathophysiology of PCOS [6].

Due to the complicated etiology and inherent endocrine dysfunction, PCOS patients are at an increased risk for the development of several comorbidities, including obesity, type 2 diabetes, non-alcoholic fatty liver disease, infertility, dyslipidemia, endometrial dysplasia, cardiovascular disorders, and psychotic disorders, such as depression and anxiety [9,10].

Due to the pathophysiological link between insulin resistance (IR) and PCOS abnormalities, insulin sensitizers have routinely been used as a treatment. Metformin (MET) is the most prevalent insulin sensitizer, typically employed in treating type 2 diabetes, and as an off-label drug in non-diabetic women with PCOS [11]. MET has been shown to impart numerous metabolic and reproductive benefits, including weight reduction, ameliorating IR and androgen levels, and restoring regular menstrual cyclicity and ovulation [12,13]. However, its use is known to be accompanied by considerable side effects, such as nausea, vomiting, and gastrointestinal discomfort [14]. The dissatisfactory compliance noted with MET prompted the discovery of innovative techniques to treat PCOS.

One of the latest advancements in the treatment of PCOS is myoinositol (MI), a naturally occurring substance that has been studied in the last decade because of its insulin-sensitizing effects and broad safety profile [15,16]. Multiple clinical trials have been carried out to evaluate MI's efficacy in addressing metabolic and reproductive symptoms of PCOS women [17,18].

Various studies have been done on MET, D-chiro inositol (DCI, a stereo-isomer of inositol, such as MI), and MI. However, there are insufficient studies that compare the efficacy of these drugs either alone or in combination in treating PCOS. For this reason, we have decided to systematically review those studies to compare these two drugs, MET and MI, for their efficacy and safety in PCOS patients.

**Figure 1:**
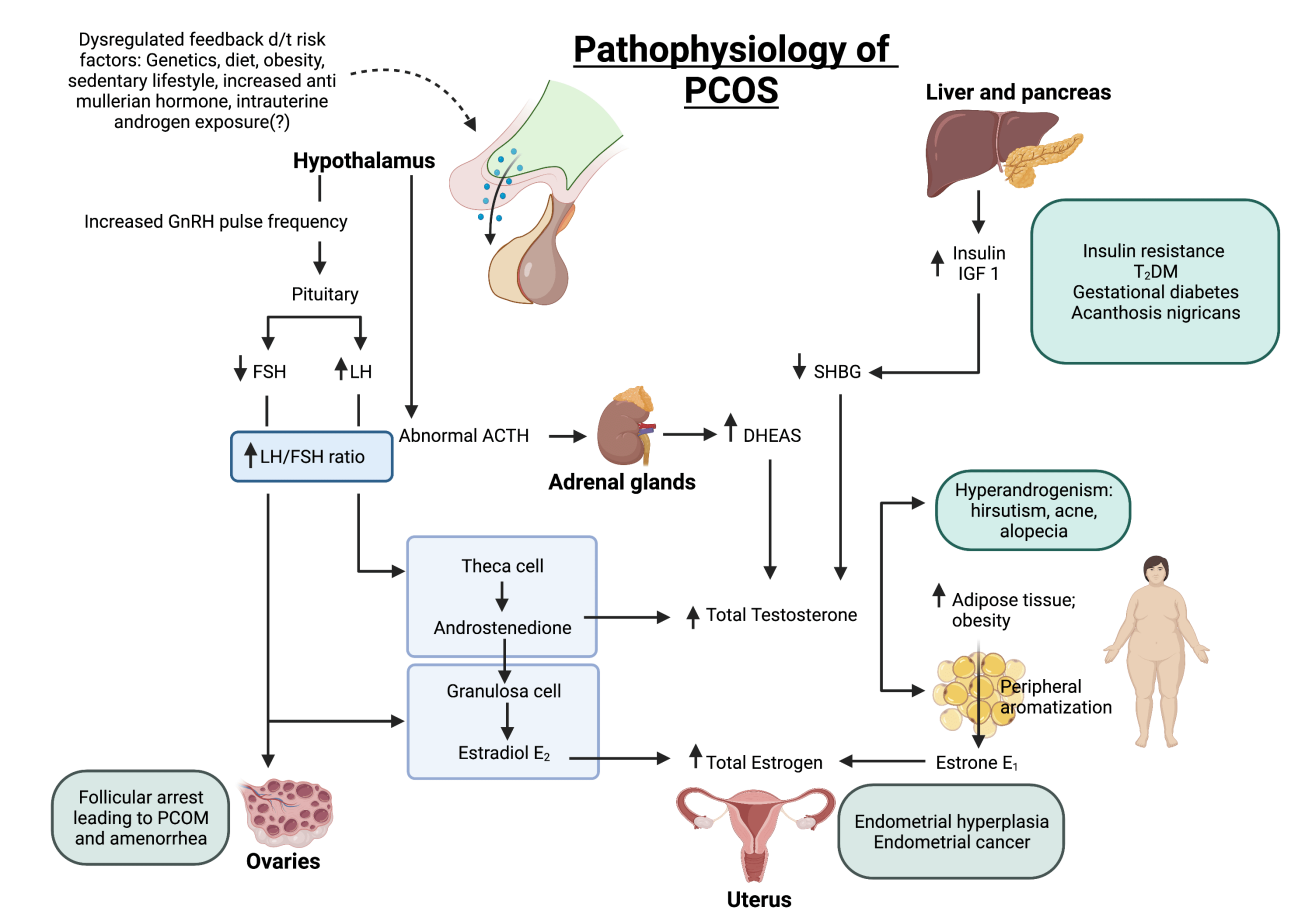
Pathophysiology of PCOS d/t: due to; GnRH: gonadotropin-releasing hormone; FSH: follicle-stimulating hormone; LH: luteinizing hormone; ACTH: adrenocorticotropic hormone; DHEAS: dehydroepiandrosterone sulfate; E1: estrone; E2: estradiol; SHBG: sex hormone-binding globulin; IGF1: insulin-like growth factor 1; T2DM: type 2 diabetes mellitus; PCOM: polycystic ovarian morphology; PCOS: polycystic ovarian syndrome. Created with BioRender.com by the authors.

Figure 1 depicts the pathophysiology of PCOS and the various clinical features that arise as a result.

## Review

Methods

We conducted and reported this systematic review in accordance with the Preferred Reporting Items for Systematic Review and Meta-Analyses (PRISMA) 2020 checklist [19].

**Figure 2:**
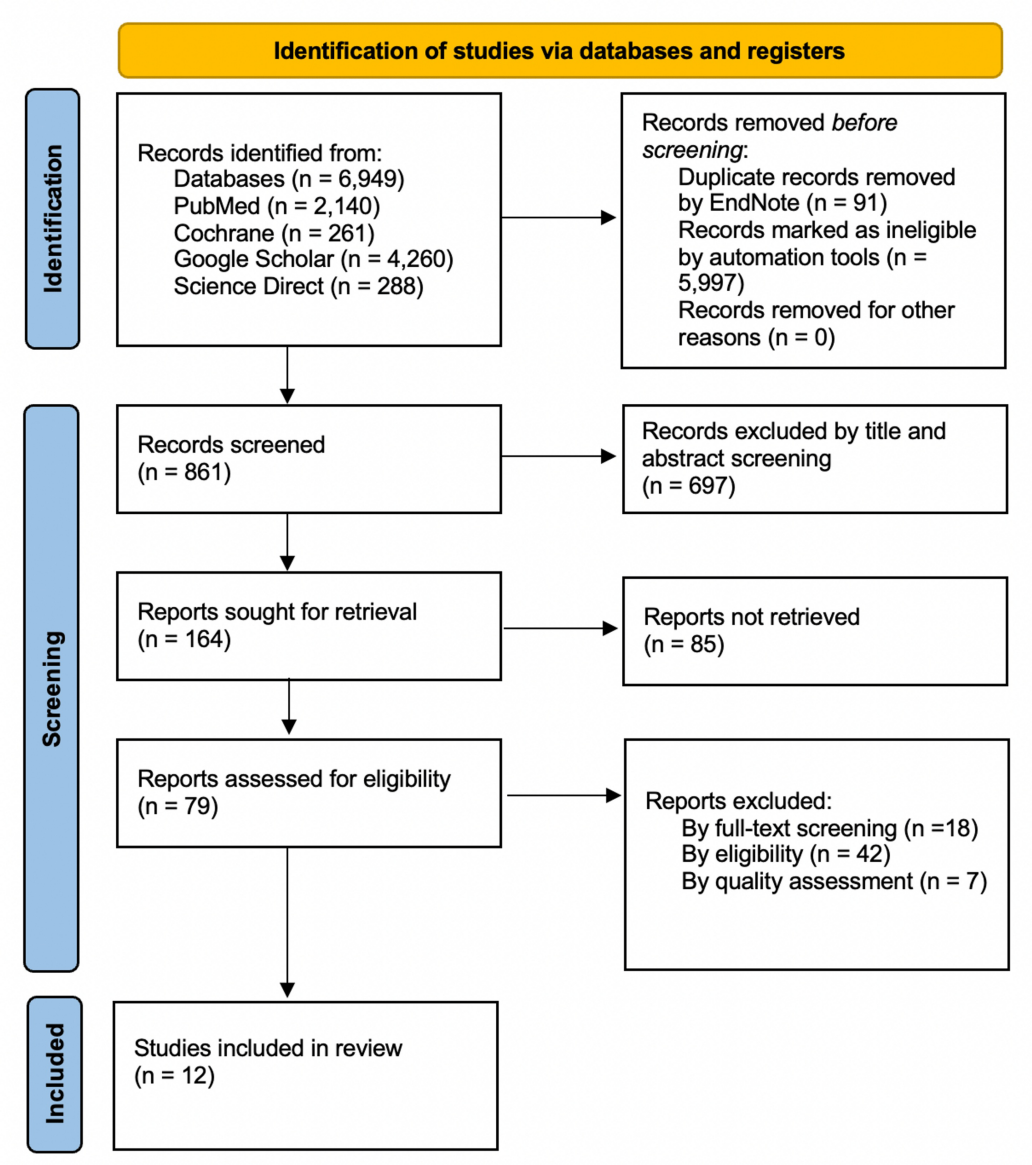
PRISMA 2020 flow diagram for new systematic reviews, which included searches of databases and registers only PRISMA: Preferred reporting items for systematic reviews and meta-analysis [19]. The figure was created by the authors.

The PRISMA flow chart is illustrated in Figure 2 [19].

Search Sources and Search Strategy

Electronic searches on PubMed, PubMed Central, Medline, Cochrane, Google Scholar, and ScienceDirect were undertaken from 18th September to 1st October 2022, and relevant articles were retrieved. A Boolean search was carried out using medical subject heading (MeSH), regular keywords, and synonyms pertinent to the subject, including "polycystic ovarian syndrome," "metformin," and "myoinositol," both alone and in combination. The selection process was further streamlined with the help of the built-in search functions of the websites. Through this process, the articles were retrieved and manually sorted to include only those pertinent to the study.

The keywords utilized in the review are shown in Table 1.

**Table 1:** Keywords employed in the study The search was conducted in October 2022. PCOS: polycystic ovarian syndrome; MeSH: medical subject headings.

Search strategy	Keywords
Regular keywords	Polycystic ovary syndrome; PCOS; Stein-Leventhal syndrome; Metformin; Inositol; Myoinositol; Efficacy; Safety
MeSH keywords	((((( "Polycystic Ovary Syndrome/drug therapy"[Majr] OR "Polycystic Ovary Syndrome/prevention and control"[Majr] OR "Polycystic Ovary Syndrome/therapy"[Majr] )) AND (( "Metformin/adverse effects"[Majr] OR "Metformin/pharmacokinetics"[Majr] OR "Metformin/poisoning"[Majr] OR "Metformin/therapeutic use"[Majr] OR "Metformin/toxicity"[Majr] ))) AND (( "Inositol/adverse effects"[Majr] OR "Inositol/pharmacokinetics"[Majr] OR "Inositol/poisoning"[Majr] OR "Inositol/therapeutic use"[Majr] OR "Inositol/therapy"[Majr] OR "Inositol/toxicity"[Majr] ))) OR ((( "Polycystic Ovary Syndrome/drug therapy"[Majr] OR "Polycystic Ovary Syndrome/prevention and control"[Majr] OR "Polycystic Ovary Syndrome/therapy"[Majr] )) AND (( "Metformin/adverse effects"[Majr] OR "Metformin/pharmacokinetics"[Majr] OR "Metformin/poisoning"[Majr] OR "Metformin/therapeutic use"[Majr] OR "Metformin/toxicity"[Majr] )))) OR ((( "Polycystic Ovary Syndrome/drug therapy"[Majr] OR "Polycystic Ovary Syndrome/prevention and control"[Majr] OR "Polycystic Ovary Syndrome/therapy"[Majr] )) AND (( "Inositol/adverse effects"[Majr] OR "Inositol/pharmacokinetics"[Majr] OR "Inositol/poisoning"[Majr] OR "Inositol/therapeutic use"[Majr] OR "Inositol/therapy"[Majr] OR "Inositol/toxicity"[Majr] )))

Inclusion and Exclusion Criteria

Our review included full-text peer-reviewed papers written in English that were published between 2012 and 2022 with an available abstract and complete, unrestricted text. The studies included only females of all ages from all over the world, representing individuals diagnosed with PCOS according to the Rotterdam, the National Institutes of Health (NIH), or the Androgen Excess and Polycystic Ovary Syndrome (AE-PCOS) society criteria [20, 21]. The participants of the studies received treatment with either MET or MI (used alone or in combination with either D-chiro inositol or folic acid) or both.

Any unpublished articles, studies without access to the full text, studies without a method section, review papers, animal or cell culture studies, case reports, grey literature, and research published before 2012 were excluded. We also excluded studies involving pregnant or lactating patients, other endocrinopathies causing similar symptoms, patients already on other drug treatments for PCOS, treatments combined with other drugs or supplements (excluding D-chiro inositol and folic acid), papers that covered fertility induction medication and pregnancy as a primary outcome, and studies with a low-quality appraisal.

Data Extraction

The selection process was carried out by two independent reviewers. Search queries were developed for PubMed, PubMed Central, Medline, Cochrane, ScienceDirect, and Google Scholar, which helped to streamline the identification of studies. Records deemed ineligible were deleted using the database's automated tools. Subsequently, duplicates were deleted using the EndNote program. The screening process involved reading titles and abstracts of articles and selecting those that could be relevant to our study. A manual search was conducted to locate the reports' full text. Articles for which full texts could not be retrieved were excluded. Ultimately, 861 records were examined, and 142 articles were included for full-text screening, eligibility, and quality assessment.

Quality Assessment and Analysis of the Studies

We assessed the quality of the articles using quality assessment measurement tools. Cochrane risk of bias was employed for the quality assessment of randomized controlled trials (RCTs) [22]. For systematic reviews and meta-analysis, the assessment of multiple systematic reviews (AMSTAR 2) checklist was used [23]. For non-randomized clinical trials and observational studies, the Joanna Briggs Institute (JBI) critical appraisal tool was utilized [24]. Only studies where both reviewers had a 95% accord and had more than 60% in terms of low-risk bias have been included. Any differences in opinions were resolved through discussion between the other authors and, if necessary, consultation with our mentor. This review has a total of 12 articles.

The risk assessment summary for selected studies is shown in Tables 2-4.

**Table 2:** Cochrane risk of bias (R.O.B.2) tool for randomized controlled trials Cochrane risk of bias tool [22].

Study name and year	Risk of bias arising from the randomization process	Risk of bias due to deviations from the intended interventions	Missing outcome data	Risk of bias in the measurement of the outcome	Risk of bias in the selection of the reported result	Overall bias
Agarwal et al. (2021) [25]	Low risk	Unclear	Low	Low	Low	Low
Angik et al. (2015) [26]	Low risk	Low	Low	Low	Low	Low
Anu et al. (2021) [27]	High	High	High	Unclear	Low	High
Bahadur et al. (2021) [28]	High	High	Low	Low	Low	Low
Chinthana (2019) [29]	High	High	High	High	Low	High
Majeed et al. (2021) [30]	Unclear	unclear	Low	High	Low	High
Jaura et al. (2020) [31]	Unclear	Unclear	Unclear	High	Low	High
Nabi et al. (2018) [32]	Unclear	High	Low	Low	Low	Low
Nehra et al. (2016) [33]	High	High	Low	Low	Low	Low
Nehra et al. (2017) [34]	High	High	Low	Low	Low	Low
Nehra et al. (2017) [35]	High	High	Low	Low	Low	Low
Duraisingam et al. (2020) [36]	Unclear	High	Unclear	High	Low	High
Shivani et al. (2021) [37]	Unclear	High	Low	High	Low	High
Thakur et al. (2020) [38]	Unclear	High	Low	High	Low	High
Thalamati (2019) [39]	High	High	Low	Low	Low	Low

**Table 3:** AMSTAR checklist for quality appraisal of systematic reviews AMSTAR = Assessment of multiple systematic reviews [23].

AMSTAR checklist	Facchinetti et al. [40]	Azizi Kutenaei et al. [41]	Zhang et al. [42]
1.	Yes	Yes	Yes
2.	Yes	Yes	Yes
3.	Yes	No	No
4.	Yes	Yes	Yes
5.	Yes	Yes	Yes
6.	Yes	Yes	No
7.	Yes	No	No
8.	Yes	Partial yes	Partial yes
9.	Yes	No	Yes
10.	No	No	No
11.	Yes	Yes	Yes
12.	No	No	No
13.	Yes	Yes	Yes
14.	Yes	Yes	Yes
15.	No	Yes	Yes
16.	Yes	Yes	Yes

**Table 4:** JBI checklist for quality appraisal of quasi-experimental studies (non-randomized experimental studies) JBI = Joanna Briggs Institute [24].

JBI checklist	Awalekar et al. [43]
1.	Yes
2.	Yes
3.	Yes
4.	No
5.	Yes
6.	No
7.	Yes
8.	Yes
9.	Yes

Results

Literature Search

Our search strategy yielded 164 possibly relevant articles, out of which 79 had full-text availability. A further 42 papers were dismissed owing to noncompliance with the eligibility criteria. Another 18 papers were excluded from the research on full-text screening. An additional seven reports were deemed ineligible using quality assessment tools. In the end, 12 articles were accepted.

Study Characteristics

The systematic review includes eight RCTs, three meta-analyses, and one non-randomized clinical trial (NN-RCT). All of the articles employed MET and MI as interventions for treating PCOS. The 12 papers were published between 2015 and 2022, and study participants originated from three countries, i.e., India [25,26,28,32-35,39-43], Iran [40-42], and Italy [40-42]. Treatment duration ranged between 12 weeks and six months. Eight papers studied the efficacy of both interventions on anthropometric measures, such as weight, waist-to-hip ratio (WHR), waist circumference, hip circumference, and body mass index (BMI). Seven papers incorporated studies on the influence of the interventions on clinical outcomes, including menstrual cycle abnormalities, hirsutism, acne, and ovarian morphology on ultrasonography (USG). Ten papers study their effect on the hormonal profile, which include serum testosterone, luteinizing hormone (LH), follicle-stimulating hormone (FSH), dehydroepiandrosterone (DHEA), androstenedione, sex hormone binding globulin (SHBG), prolactin (PL), and progesterone. The effect on biochemical measures, such as fasting blood sugar (FBS), post-meal blood sugar (PMBS), insulin, glucose-to-insulin ratio, lipid profile, and the Homeostatic Model Assessment for Insulin Resistance (HOMA-IR), was analyzed in 10 papers. Adverse events (AE) were assessed in six papers. It is crucial to note that, currently, there is no single highly effective treatment for PCOS. On account of the complex and multifaceted nature of the syndrome, it is best-addressed case by case with an individually tailored treatment plan.

The essential characteristics of the incorporated studies are highlighted (Table 5) below.

**Table 5:** General characteristics of included studies RCT: randomized controlled trial; NN-RCT: non-randomized clinical trial; AMSTAR: Assessment of multiple systematic reviews; JBI: Joanna Briggs Institute; MI: myoinositol; MET: metformin; DCI: D-chiro-inositol; BMI: body mass index; WHR: waist-to-hip ratio; FBS: fasting blood sugar; PMBS: post-meal blood sugar; LH: luteinizing hormone; FSH: follicle-stimulating hormone; AFC: antral follicle count; HOMA-IR: homeostatic model of assessment for insulin resistance; AE: adverse event; PL: prolactin; USG: ultrasonogram; IR: insulin resistance; SHBG: steroid hormone binding globulin; DHEA: dehydroepiandrosterone; 17-OH-P: 17-hydroxy progesterone

Author	Year of Publication	Study Design	Quality Appraisal Tool	Number (N^o ^) of Subjects/Studies	Intervention	Duration	Outcomes Measured
Angik et al. [26]	2015	RCT	Cochrane risk of bias assessment tool	(N^o ^= 100) MI: 50 MET: 50	MI 1g b.i.d Vs. MET 500mg b.i.d	Six months	Menstrual abnormalities, hirsutism, acne, BMI, WHR, FBS, PMBS, post-meal insulin, testosterone, LH, LH/FSH ratio, mean ovarian volume, AFC, fasting insulin, HOMA-IR, pregnancy, AE
Awalekar et al. [43]	2015	NN-RCT	JBI checklist	(N^o^ = 67) MI: 32 MET: 35	MI 2g b.i.d, Folic acid 5mg/day Vs. MET 500mg t.i.d	Three months	BMI, HOMA-IR, LH/FSH ratio
Nehra et al. [33]	2016	RCT	Cochrane risk of bias assessment tool	(N^o ^= 60) MI: 30 MET: 30	MI 1g b.i.d Vs. MET 500mg t.i.d	24 weeks	Regularity of menstrual cycle, hirsutism, weight changes, AE
Nehra et al. [34]	2017	RCT	Cochrane risk of bias assessment tool	(N^o ^= 60) MI: 30 MET: 30	MI 1g b.i.d Vs. MET 500mg t.i.d	24 weeks	body weight, BMI, waist circumference, hip circumference, WHR
Nehra et al. [35]	2017	RCT	Cochrane risk of bias assessment tool	(N^o ^= 60) MI: 30 MET: 30	MI 1g b.i.d Vs. MET 500mg t.i.d	24 weeks	Insulin, FSH, LH, LH/FSH ratio, testosterone, HOMA-IR, glucose/insulin ratio, lipid profile
Nabi et al. [32]	2018	RCT	Cochrane risk of bias assessment tool	(N^o^ = 63) MI: 32 MET: 31	MI 2g/day Vs. MET 500mg b.i.d	16 weeks	Weight, BMI, WHR, FBS, PMBS, fasting insulin, LH, LH/FSH ratio, testosterone, PL, hirsutism, progesterone, USG characteristics, AE
Facchinetti et al. [40]	2019	Meta-analysis	AMSTAR 2 checklist	Six RCTs with (N^o ^= 355) MI: 177 MET: 178	MI 2-4g/day Vs. MET 1.5-2g/day	12-24 weeks	Insulin, HOMA-IR, testosterone, androstenedione, SHBG, BMI, AE
Thalamati [39]	2019	RCT	Cochrane risk of bias assessment tool	(N^o ^= 200) MI: 100 MET: 100	MI 550mg + DCI 13.8mg b.i.d Vs. MET 500mg t.i.d	24 weeks	Menstrual cycle regulation, hirsutism, insulin, glucose, DHEA, LH/FSH ratio, testosterone, HOMA-IR, glucose/insulin ratio
Agarwal et al. [25]	2021	RCT	Cochrane risk of bias assessment tool	(N^0 ^= 50) MI: 25 MET: 25	MI 1g b.i.d Vs. MET 850mg b.i.d	Six months	menstrual abnormality, acne, hirsutism, USG characteristics, LH, FSH, LH/FSH ratio, testosterone, SHBG, lipid profile, FBS, PMBS, fasting insulin, post-meal insulin, glucose/insulin ratio, HOMA-IR, AE
Bahadur et al. [28]	2021	RCT	Cochrane risk of bias assessment tool	(N^o ^= 72) MI+MET: 36 MET: 36	MI 550mg+DCI 150mg+MET 500mg b.i.d Vs. MET 500mg b.i.d	Six months	menstrual cycle irregularity, hirsutism, acne, waist circumference, hip circumference, WHR, BMI, lipid profile, FBS, PMBS, fasting and post-meal insulin, HOMA-IR, LH/FSH ratio, serum testosterone, DHEA
Azizi Kutanaei et al. [41]	2021	Meta-analysis	AMSTAR 2 checklist	Nine studies: seven RCTs, 1 NN-RCT, and one retrospective study (N^o^ = 684) MI: 331 MET: 353	Not included	12 weeks- six months	Menstrual irregularity, LH, FBS, FSH, LH/FSH, HOMA-IR, testosterone, prolactin, DHEA, 17-OH-P (17-hydroxy progesterone), androgen, estradiol, SHBG, ovarian volume, insulin
Zhang et al. [42]	2022	Meta-analysis	AMSTAR 2 checklist	Nine RCTs with (N^o^ = 612) MI: 306 MET: 306	MI 1-4g/day +/- 40-200 mcg folic acid/day Vs. MET 500-1500mg/day +/- folic acid 200mcg/day	three-six months	BMI, WHR, androstenedione, testosterone, DHEA, SHBG, lipid profile, FBG, fasting insulin, HOMA-IR, AE

The results of the outcome variables of included studies are displayed (Table 6) below.

**Table 6:** A highlight summary of involved studies with outcomes of interest and results MI: myoinositol; MET: metformin; DCI: D-chiro-inositol; PCOS: polycystic ovarian syndrome; mFG: modified Ferriman-Gallwey; BMI: body mass index; WHR: waist-to-hip ratio; FBS: fasting blood sugar; PMBS: post-meal blood sugar; LH: luteinizing hormone; FSH: follicle-stimulating hormone; AFC: antral follicle count; HOMA-IR: homeostatic model of assessment for insulin resistance; AE: adverse event; PL: prolactin; USG: ultrasonogram; IR: insulin resistance; SHBG: steroid hormone binding globulin; DHEA: dehydroepiandrosterone; 17-OH-P: 17-hydroxy progesterone; TC: total cholesterol; HDL: high-density lipoprotein; LDL: low-density lipoprotein; PCOM: polycystic ovarian morphology

Study name and year	Results	Conclusions
Angik et al. (2015) [26]	Overall, 37.73% achieved regular cycles, 28.57% with MI, and 48% with MET. The decrease in the mean mFG score of hirsutism and acne was statistically significant in both groups; however, the reduction was not substantial between the two groups. Similar outcomes were noted in BMI, WHR, FBS, PMBS, post-meal insulin, testosterone, LH, LH/FSH ratio, mean ovarian volume, and antral follicle count (AFC). The mean fasting insulin decrease in MI was statistically significant; in the MET group, the decline was insignificant. The HOMA-IR index decrease was substantial in the MI group, but the decrease was not significant in the MET group. Between the two groups, the HOMA-IR decrease was substantial. In total, 72% of patients experienced the side effects of treatment with MET; 2% of patients had lactic acidosis; 38% had generalized weakness; 32% had nausea; and 28% had no side effects. On the other hand, only 16% of patients in the MI group experienced side effects - 14% had menorrhagia and 2% had nausea.	MET is effective in ameliorating metabolic and hormonal parameters. MI has the advantage of decreasing insulin resistance in addition to improving the above parameters. MI is also well tolerated and has better patient compliance than MET.
Awalekar et al. (2015) [43]	BMI: In the MET group, the mean BMI reduced from 29.64 to 27.13. In the MI group, the mean BMI decreased from 25.40 to 24.40. HOMA-IR index: For MET, the mean HOMA-IR index reduced from 25.85 to 15.2. For MI, the change was from 23.74 to 23.8; which is not statistically substantial. LH/FSH ratio: For MET, it reduced from a mean of 2.56 to 1.7. For MI, the reduction was from a mean of 2.32 to 2.10.	Both MET and MI effectively reduce the BMI and LH/FSH ratio, but only MET showed statistically significant improvement in the HOMA-IR index. So, in this study, MET is better as a treatment for insulin resistance.
Nehra et al. (2016) [33]	Overall, 88% attained regular cycles, 87% with MI, and 90% after MET treatment. The results were comparable in both groups. In both groups, there was a statistically significant decrease in patients having PCOM at the end of 24 weeks compared to baseline. For the MI cohort, the percentage decreased from 83% to 13%. The metformin group's rate fell from 80% to 17%. At the end of 24 weeks, there was more significant progress in the number of patients having normal ovaries on USG compared to baseline values with MI than MET (70% vs. 63%). A comparable response was noted with both drugs in reducing the mFG score. Both groups displayed a statistically significant weight reduction compared to baseline values. There was more weight reduction with MI than with MET, but the difference was not statistically significant. There was slightly more improvement in BMI with MI than with MET, but the difference was not statistically significant. In the MI Group, the improvement in acne was 31%. In the MET group, the improvement was 32%. The incidence of various AEs was higher in the MET group than the MI group, apart from menorrhagia, which was observed only with MI and dizziness, which was comparable in both groups. The MET cohort displayed a greater incidence of nausea, diarrhea, abdominal cramps, flatulence, and generalized weakness.	MI and MET were safe and efficacious in treating patients suffering from PCOS. Both treatments improved the regularity of the menstrual cycle and androgenic features such as acne, hirsutism, PCOM, and BMI. In comparison, the overall results of MI were comparable with that of MET. However, in terms of safety, MI was better tolerated. Therefore, MI can be a new addition to the treatment of PCOS with similar efficacy and better safety profile.
Nehra et al. (2017) [34]	Weight: With MI, the mean decrease in weight was from 63.96 to 61.20, with a total reduction of 2.76. The mean reduction in weight was from 63.58 to 60.86, with a total decrease of 2.72 in the MET group. There was no statistically significant decrease between the two groups. BMI: In the MI group, the BMI decreased by 1.14, while in the MET group, the BMI decreased by 1.13 MI was slightly improved compared to MET, but the difference was not statistically significant. WHR: WHR decreased by 0.01 with MI, whereas, in the MET group, no decrease in WHR was observed at the end of 24 weeks. Comparing values between MET and MI was not statistically significant.	MET and MI improved anthropometric parameters in PCOS. Both drugs were found to be equally efficacious.
Nehra et al. (2017) [35]	A statistically substantial improvement was observed in insulin resistance, glucose/insulin ratio as well as HOMA-IR in both groups. The hormonal parameters demonstrated statistically significant improvements in both drugs concerning changes in FSH, LH, LH /FSH ratio, and testosterone levels. Lipid profiles also improved for both MI and MET. However, on comparison, the difference was not statistically significant.	There was considerable improvement in the biochemical profile of PCOS patients with both MET and MI.
Nabi et al. (2018) [32]	In the MI group, 53.1% of subjects attained cycle regularity, while in the MET group, 41.9% achieved regular cycles. The decrease in the number of follicles was more prominent with MI than with MET, while the decrease in ovarian volume was almost the same in both groups. There was a substantial drop in body weight and WHR in both groups, but between the two, it was more significant in the MI group. The reduction in BMI was greater in the MET group. However, for all three indices, the differences were not statistically significant. There were notable drops in the mFG score for both groups as well, however, the fall in the MI group was more effective than for the MET group. Fasting insulin dropped from 13.90 ± 6.88 SD to 11.66 ± 6.05 SD in the MI group and from 12.85 ± 4.46 SD to 11.78 ± 4.39 SD in the MET group. MI displayed a highly significant drop compared to MET. FBS was standard in all patients; MET had a more significant drop than MI. For PMBS, the decrease was more significant for MI. No significant results were noted for LH; however, group MET demonstrated a more significant drop in LH/FSH ratio than in Group MI. The fall in free testosterone was more effective in the MI group than in the MET subjects. The difference in serum progesterone value was more significant in MI than in MET. Only 15.6% of patients experienced AEs with MI. Menorrhagia was a complaint observed only with MI. In the MET group, 64.5% experienced AEs. Among the observed AEs, only abdominal cramps had a statistically significant p-value.	Metabolic and hormonal parameters are effectively regulated with MET. MI not only improves the above parameters but also significantly reduces insulin resistance. MI is also better tolerated. Thus, MI supplementation is beneficial in managing PCOS to improve insulin sensitivity.
Facchinetti et al. (2019) [40]	For fasting insulin, HOMA index, testosterone, androstenedione, SHBG levels, and BMI, no difference was noted between MET and MI. A moderate heterogeneity among studies for fasting insulin, HOMA index, SHBG, and BMI was found. Whereas studies on serum testosterone and androstenedione revealed no heterogeneity across studies. There was compelling evidence of a heightened risk of AEs among women receiving MET compared to those receiving MI. Subjects in the MET group were almost five times more likely to have AEs than those in the MI group. No heterogeneity among studies was found. The most recorded AEs with MET were nausea and diarrhea, with reports of a severe entity in some cases, abdominal pain, lactic acidosis, and generalized weakness. With MI, they were nausea, mild diarrhea, and menorrhagia. MET and MI displayed no differences in short-term hormone changes.	Due to its better tolerance, MI is more suitable for improving the androgenic and metabolic profiles in PCOS women.
Thalamati (2019) [39]	In the MI + DCI group, the menstrual cycle length decreased by seven days, the percentage of women with regular menstrual cycles increased by 20%, and the hirsutism score dropped by 6 points. In the MET group, the menstrual cycle length decreased by seven days, the percentage of women with regular menstrual cycles increased by 12%, and the hirsutism score dropped by 1 point. Insulin resistance in both groups demonstrated a statistically significant improvement as assessed with glucose-insulin ratio and HOMA-IR. The MI + DCI group saw an increase of 1.20 in the glucose-insulin ratio and a decrease of 1.32 in the HOMA-IR. In contrast, the MET group showed an increase of 1.03 in the glucose-insulin ratio and a decrease of 1.10 in HOMA-IR. The hormonal parameters demonstrated statistically substantial improvements in hormonal parameters as evaluated by changes in DHEA, FSH, LH, LH/FSH ratio, and testosterone levels with MI + DCI group as compared to the MET group. In the MI + DCI group, the DHEA level decreased by 76mcg/dl, and the LH/FSH ratio and testosterone decreased by 0.82 and 2.90, respectively. In contrast, in the MET group, the DHEA level decreased by 30mcg/dl, the LH/FSH ratio decreased by 0.35, and testosterone by 0.840.	Both the combination of MI and DCI and MET alone, significantly improved insulin sensitivity in PCOS women. However, the combination of MI and DCI effects better regulated the hormonal profiles (LH/FSH ratio and free testosterone) in contrast to MET.
Agarwal et al. (2021) [25]	MI demonstrated greater improvement in cyclicity. Regular cycles were seen in 68% vs. 28%, cycle length was 34 vs. 45 days, and cycles per year were 9.4 vs. 8.4 with MI and MET, respectively. Reduction in hirsutism and acne was similar. On ultrasound, ovaries achieved better morphology with MI in volume and stromal thickness. The number of follicles decreased similarly for both interventions. A significant reduction was seen in testosterone (20%) and LH/FSH ratio (12.5%) only with MI. SHBG improvement was similar in both groups. Among metabolic parameters, lipids (LDL, HDL, and total cholesterol) and blood sugars improved with MET, whereas the effect on insulinemic parameters (insulin, HOMA-IR) was similar. Lesser AEs were observed with MI.	MI is comparable to MET in improving insulin sensitivity and is equally effective in treating PCOS. It regulates menstrual cyclicity, testosterone, and ovarian morphology better than MET, whereas MET corrects lipids and blood sugar better than MI. Using either of them or both together can be made based on the patient profile. MI is a nutraceutical with lesser AEs and hence better patient compliance. The results demonstrate that it can be a reliable therapeutic in the treatment of PCOS.
Bahadur et al. (2021) [28]	Cycle irregularity was observed at baseline in all the study subjects of both groups. At follow-up, 61.1% of patients in the MET+MI+DCI group had achieved regular cycles, whereas only 36.1% of the MET group had attained regularity. In the MET+MI+DCI group, mean BMI decreased from 25.29±4.13 to 23.34±3.14 and mean WHR changed from 0.85±0.06 to 0.85±0.05. For the MET group, the mean BMI decreased from 23.43±4.76 to 23.36±4.08, and the mean WHR changed from 0.84±0.08 to 0.85±0.08. A statistically significant difference was noted in terms of mean global acne score and cycle regularity after six months of treatment in the MET+MI+DCI group. A significant difference in values of LH, LH/FSH ratio, mean cholesterol, mean HDL, mean LDL, and postprandial insulin was also observed in the MET+MI+DCI group. The hormonal parameters of mean FSH, mean testosterone and mean DHEAS demonstrated no substantial difference between the two groups. The mean triglyceride, mean fasting sugar, mean PMBS, mean fasting insulin, and HOMA-IR index also displayed no difference between both cohorts.	The results indicate that there is a promising outlook for the combined therapy of MET and MI plus DCI in women with PCOS and insulin resistance.
Kutanaei et al. (2021) [41]	LH - MET had a significantly more effective outcome on LH than MI. LH/FSH - the LH/FSH ratio in the MI group was significantly higher than that of the MET group. Serum testosterone - Substantial heterogeneity was detected among the studies. The level of testosterone in the MET group was significantly higher than that observed in the MI group. In other words, MI was more effective than MET. Prolactin - There was evidence of a high heterogeneity among the studies. The level of prolactin in the MI group was significantly higher than that of the MET group. DHEA - DHEA in the MET group was significantly higher than that of the MI group. Therefore, MI was more effective than MET in modulating hyperandrogenism. 17-OH-P- MET was more effective than MI in reducing the 17-OH-P. Other characteristics - Other indices, such as FBS, FSH, estradiol, ovarian volume, and insulin, were estimated to be lower in the MET group than in the MI group. However, there was no statistically significant difference between the effectiveness of the two treatments.	MI would probably be an effective alternative treatment for PCOS patients undergoing conventional drug therapies. Also, based on the current evidence, MI can be advantageous in treating PCOS hyperandrogenism.
Zhang et al. (2022) [42]	Impact on androgen-associated hormones - No significant differences were observed between MI and MET in both total testosterone and SHBG. Impact on body fat - For both BMI and WHR, no significant differences were observed between MI and MET. Impact on glucose metabolism and lipid metabolism parameters - No significant differences were seen in fasting insulin, FBG, HOMA-IR, TC, HDL, and LDL between MI and MET. Results showed that MI could lower triglyceride levels compared to MET. AEs - Most subjects experienced no side effects with MI; however, participants in the MET group encountered several AEs, such as lactic acidosis, weakness, and gastrointestinal symptoms.	Compared to MET, MI may be better at lowering the triglycerides of patients with PCOS. Additionally, using MIs under a judicial dosage range could largely avoid the AEs of MET use.

Discussion

To determine and compare the safety and efficacy of MET and MI in PCOS, we addressed the outcomes of anthropometric, hormonal, biochemical, and clinical indices as indicators of efficacy and the development of AEs as a gauge of their safety profile.

Efficacy of MET and MI

Menstrual regulation: Overall, there was a comparable improvement in menstrual regularity with both modalities in studies by Angik et al., Nehra et al., Nabi et al., and Kutanaei et al. [26,33,32,41]. However, in a study by Thalamati et al., the MET+MI+DCI group achieved a higher success rate of 20% vs. 12% in the MET group [39]. In another study by Agarwal et al., better outcomes were seen in the MI group compared to the MET group (68% vs. 28%) [25]. Bahadur et al. found that 61.1% of patients in the MET+MI+DCI group had attained regular cycles compared to only 36.1% in the MET group [28]. Overall, the results demonstrate that, either alone or in combination with other modalities, MI is beneficial in menstrual cycle regularization.

Ovarian morphology: Five papers addressed changes in ovarian morphology. Both MI and MET are effective in improving ovarian morphology. Angik et al. observed a similar decrease in mean ovarian volume and AFC in both groups [26]. Similarly, Nehra et al. noted an equivocal reduction in polycystic ovarian morphology (PCOM) in both treatment groups [33]. They also concluded that there was a more substantial improvement in the number of subjects having normal ovaries on USG compared to baseline values with MI than MET (70% vs. 63%). In the paper by Nabi et al., the reduction in the number of follicles was considerably more with MI than with MET, while the decrease in ovarian volume was almost the same in both groups [32]. Likewise, on ultrasound, Agarwal et al. found that ovaries achieved better morphology with MI in volume and stromal thickness [25]. Still, the number of follicles decreased similarly for both interventions. Ovarian volume was estimated to be lower in the MET group than in the MI group; however, the difference was not statistically significant according to the results from the study by Kutanaei et al. [41]. MI seems to play a substantial role in normalizing ovarian morphology and recent studies suggest it could be a key factor in improving oocyte and embryo quality.

Anthropometric measures: Statistically significant declines were seen in BMI in both MI and MET, but the difference between the two was not substantial in studies by Angik et al., Awalekar et al., Nehra et al., Nabi et al., Facchinetti et al., and Zhang et al. [26,43,34,32,40,42]. Nabi et al. found that the fall in mean BMI was more remarkable in the MET group [32]. This is in contrast to the study by Bahadur et al., in which they noted a more significant decrease in the MET+MI+DCI group [28]. This can likely be attributed to the difference in interventions utilized as the study measured MET against the combination of MET, MI, and DCI.

Nehra et al. observed that there was a greater reduction in weight with MI as compared to MET, but the difference was not statistically significant [34]. Nabi et al. also noted that, although the drop in body weight was significant in both groups, it was more significant in the MI group [32]. However, again, the difference between the two was not statistically significant.

Similar observations were made in the WHR. Angik et al., Nabi et al., and Zhang et al. all noted significant reductions in the WHR, but the difference between MET and MI groups was not statistically significant [26,32,42]. The study by Bahadur et al. displayed no considerable change in WHR in the groups [28].

Traditionally, the first-line treatment for obese and overweight PCOS patients included lifestyle modifications, such as a hypocaloric diet and physical activity. If patients were found to be resistant to weight loss through lifestyle interventions, then MET would be prescribed as adjuvant therapy. The results of this review show that MI may be considered an equally effective alternative.

Hirsutism and acne: The studies incorporated in this review assessed hirsutism using the mFG score. Most studies described using the global acne score in grading acne. Angik et al. found that the decrease in the mean modified Ferriman-Gallwey (mFG) score of hirsutism and acne was statistically significant in both groups; however, the change was not significant between the two groups [26]. Nehra et al. also noted a comparable response with both drugs in reducing mFG scores [33]. In the MI group, the improvement in acne was 31%. In the MET group, the improvement was 32% [33]. In the study by Nabi et al., it was observed that the mFG score was reduced from 4.66 ± 4.06 SD to 3.56 ± 3.29 SD in the MI group and from 4.94 ± 4.05 SD to 3.87 ± 3.24 SD in the MET group; the fall in MI was more significant than MET, but the difference was not statistically significant [32]. Thalamati found that the hirsutism score decreased by one point in the MET group and six points in the MI+DCI group [39]. Statistically, a significant difference was observed in the mean global acne score of the MET+MI+DCI group's study by Bahadur et al. [28].

Hirsutism and acne are both distressing symptoms for PCOS patients. Currently, these manifestations are addressed through the use of oral contraceptives and androgen blockers, such as spironolactone, and are accompanied by their share of side effects. As the results demonstrate, the use of MI as a natural and safe alternative could be explored further.

Hormonal parameters: Angik et al. observed a significant and comparable decrease in testosterone, LH, and LH/FSH ratio [26]. Awalekar et al. and Nehra et al. also noted significant reductions in the LH/FSH ratio for both interventions [35,43]. In addition, Nehra et al. demonstrated a significant reduction in testosterone for both treatment groups [35]. Nabi et al. recorded that results for LH were not significant; however, the LH/FSH ratio fall was more marked in the MET group than in the MI group. The fall in free testosterone for MI was more significant than for MET. Changes in serum progesterone value were more effective with MI than with MET [32]. No difference between MET and MI was found in testosterone, androstenedione, and SHBG levels in the study conducted by Facchinetti et al. [40]. Thalamati et al. observed statistically significant improvements in hormonal parameters as assessed by changes in DHEA, FSH, LH, LH/FSH ratio, and testosterone levels with MI and DCI groups as compared to the MET group. In the MI + DCI group, the DHEA level decreased by 76 mcg/dl, and the LH/FSH ratio and testosterone decreased by 0.82 and 2.90, respectively. In contrast, in the MET group, the DHEA level dropped by 30 mcg/dl, the LH/FSH ratio decreased by 0.35, and testosterone by 0.840 [39]. Agarwal et al. noted a significant reduction in testosterone (20%, p=0.005) and LH/FSH ratio (12.5%, p=0.02) only with MI. SHBG improvement was similar in both groups [25]. Bahadur et al. described a significant difference in values of the LH and LH/FSH ratio in the MET+MI+DCI group. No significant difference was seen between the two groups regarding mean FSH, mean testosterone, and mean DHEAS [28]. The analysis by Kutanaei et al. found that both supplements improved estradiol and SHBG. However, the LH level, LH/FSH ratio, and prolactin levels were only enhanced by MET. MET was also more effective than MI in reducing the 17-OH progesterone. A significant decrease in the DHEA and testosterone levels, as circulating androgens, was observed in PCOS patients who received MI compared to those who received MET [41]. No significant differences between MI and MET were observed in the improvement of testosterone and SHBG in the study by Zhang et al. [42].

Both MI and MET have demonstrated their effectiveness in ameliorating the hormonal imbalances associated with PCOS. Current practice involves the use of oral contraceptives for hormonal regulation; however, given the multiple side effects associated with its use, alternate therapies such as MI should be explored as a possibility.

Dysglycemia: Angik et al. observed a significant and comparable decrease in FBS, PMBS, and post-meal insulin. The mean fasting insulin decrease in MI was statistically significant, while in the MET group, it was not. The HOMA index displayed a substantial reduction in the MI group, whereas the decrease was not significant in the MET group [26]. In contrast, Awalekar et al. found that the mean HOMA index reduced from 25.85 to 15.21 (p=0.000) for the MET group and was highly significant statistically, but the reduction for MI was not [43]. Nehra et al. observed that, in both groups, there was a statistically significant improvement in insulin resistance. With MI and MET, the values for glucose/insulin ratio improved from 6.77±0.99 to 7.87±1.03 and from 5.5±0.42 to 6.90±0.47. The HOMA index values decreased from 4.18±0.4 to 2.88±0.27 and from 4.38±0.43 to 2.99±0.29 in MI and MET, respectively [35]. According to Nabi et al., the reduction in fasting insulin was highly significant for MI compared to MET. FBS was standard in all patients. However, the decrease for MET was more effective than for MI. For PMBS, fall was more notable for MI [32]. Facchinetti et al. observed no difference between MET and MI in fasting insulin and HOMA index [40]. In the study by Thalamati et al., insulin resistance showed a statistically significant improvement as assessed with glucose-insulin ratio and HOMA-IR in both groups. The glucose-insulin ratio increased by 1.20, and HOMA-IR decreased by 1.32 in the MI+DCI group. Meanwhile, in the MET group, the glucose-insulin ratio increased by 1.03, and HOMA-IR decreased by 1.10 [39]. Agarwal et al. noted that the blood sugars improved with MET, whereas the effect on insulinemic parameters (insulin, HOMA-IR) was similar [25]. Bahadur et al. documented a significant difference in values of postprandial insulin (p=0.005) in the MET+MI+DCI group. No significant difference was seen between the two groups regarding mean fasting and PMBS, fasting insulin, and HOMA-IR index [28]. Kutanaei et al. demonstrated that FBS and insulin were estimated to be lower in the MET group than in the MI group. However, there was no statistically significant difference between the effectiveness of the two treatments [41]. Zhang et al. observed no significant differences in MET and MI groups for fasting insulin [42].

Lipid profile: According to Nehra et al., the lipid profile displayed an improvement in both groups. However, in the comparison, no statistically significant difference was observed [35]. On the other hand, Agarwal et al. found that the lipids (total cholesterol, high-density lipoprotein, low-density lipoprotein) improved with MET [25]. Bahadur et al. noted a significant difference in mean cholesterol, mean high-density lipoprotein, and mean low-density lipoprotein values in the MET+MI+DCI group. No significant difference between the two groups was seen in mean triglyceride [28]. The study by Zhang et al. showed that MI could lower triglyceride levels compared to MET. For total cholesterol, no significant differences were observed between MI and MET. High-density lipoprotein and low-density lipoprotein showed no significant differences [42].

Side Effects of MET and MI

The studies in our review reported superior safety profiles for MI compared to MET in terms of AEs, such as lactic acidosis, generalized weakness, nausea, menorrhagia, dizziness, diarrhea, abdominal cramps, and flatulence. Angik et al., Nehra et al., Nabi et al., Facchinetti et al., Agarwal et al., and Zhang et al. all reported a higher incidence of AEs with MET when compared to MI [26,33,32,40,25,42]. MET treatment's most reported side effects were generalized weakness and gastrointestinal side effects, such as nausea, abdominal cramps, diarrhea, and flatulence. The only reported AE with MI were nausea, dizziness, and menorrhagia; however, the incidence was very low. Hence, it can be concluded that MI is better tolerated and has a broader safety profile compared to MET. This also results in better patient compliance with MI.

Limitations

There are certain limitations to our study, among which the primary limitation was a dearth of high-quality clinical studies comparing the treatment of PCOS with MET and MI. It is necessary to consider the biases of the included studies as they may have influenced the accuracy of the results. Certain biases, such as the lack of well-designed controlled trials, a lack of double blinding, small sample size, variations in doses and combinations of the drugs, short study durations, and the use of various criteria for describing PCOS patients, can influence the effects and reported results of the study.

## Conclusions

Our systematic review revealed that MI could be an effective alternative or adjunctive treatment for PCOS patients undergoing conventional drug therapies. MI, a natural nutraceutical and an insulin sensitizer, is comparable to MET in treating PCOS for clinical, hormonal, lipid, glycemic, and insulinemic benefits. Additionally, MI treatment has minimal side effects and better tolerance than MET treatment. Given the increased rate of prevalence of PCOS worldwide, there is potential for further clinical studies with bigger sample sizes, longer study duration, different dosages of drugs, and studies employing a combination of other medications used in the standard treatment of PCOS.
