# Correction: Myoinositol Versus Metformin in the Treatment of Polycystic Ovarian Syndrome: A Systematic Review

**DOI:** 10.7759/cureus.c303

**Published:** 2025-09-23

**Authors:** Ranita Bodepudi, Saniya Seher, Shenel A Khan, Sonya Emmanuel, Vivig Shantha Kumar, Resheek Nerella, Basim Shaman Ameen, Dev Patel, Jabez David John, Safeera Khan

**Affiliations:** 1 Medicine, California Institute of Behavioral Neurosciences & Psychology, Fairfield, USA; 2 Internal Medicine, Lokmanya Tilak Municipal Medical College and General Hospital, Mumbai, IND

This article has been corrected to fix the following typo:

In the second sentence of the paragraph beneath the 'Literature Search' heading, "49" was corrected to "42" in the following sentence: "A further 42 papers were dismissed owing to noncompliance with the eligibility criteria."

